# Dynamic morphological plasticity in response to emergence timing in *Abutilon theophrasti* (Malvaceae)

**DOI:** 10.1002/pei3.10084

**Published:** 2022-06-18

**Authors:** Shu Wang, Dao‐Wei Zhou

**Affiliations:** ^1^ College of Forestry, Forest Ecology Research Center Guizhou University Guiyang China; ^2^ Northeast Institute of Geography and Agroecology, Chinese Academy of Sciences Changchun China

**Keywords:** biomass allocation, germination time, growth period, leaf growth, life cycle, morphological plasticity, reproduction

## Abstract

Selections on emergence time might be conflicting, suggesting the existence of the optimal emergence time for plants. However, we know little about this and how morphological plasticity contributes to the strategies of plants in response to emergence timing. To better understand this issue from a dynamic perspective, we conducted a field experiment by subjecting plants of *Abutilon theophrasti* to four emergence treatments (ET1 ~ ET4) and measuring a number of mass and morphological traits on them at different growth stages (I ~ IV). On day 50, 70, and/or final harvest, among all ET treatments, plants germinated in late spring (ET2) performed the best in total mass, spring germinants (ET1) and ET2 performed better in stem allocation, stem, and root diameters than later germinants (ET3 and ET4); summer germinants (ET3) had the highest reproductive mass and allocation, while late‐summer germinants (ET4) had the greatest leaf mass allocation, with greater or canalized leaf number, and root length traits than others. Plants that emerged in late spring can maximize their growth potential, while those with either advanced or delayed emergence are still capable of adaptation via allocation and morphological plasticity. Early germinants (ET1 and ET2) preferred stem growth to leaf and reproductive growth, due to sufficient time for reproduction in the growth season. With limited time for growth, plants that emerged late may prefer to quicken leaf growth (indicated by increased leaf mass allocation and leaf number) at the cost of stem or root growth for the complete life cycle, reflecting both positive and negative effects of delayed emergence.

## Graphical abstract

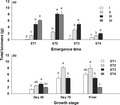

## 1 INTRODUCTION

A fundamental goal in evolutionary ecology is to understand the timing of life history in variable environments (Gremer et al., 2020). Seedling emergence, as one of the crucial events in the life cycle of plants, often determines subsequent plant performance and success (Harper, 1977; Weiner, 1988). Many annual herbaceous plants can germinate within a wide range of periods, and subsequently face contrasting environmental circumstances, which may be of great complicacy. The timing of emergence determines the environmental cues plants expose to, such as day length, temperature and water availability, and interactions with other individuals and species (Donohue et al., 2010; Lortie & Turkington, 2002; Verdu´ & Traveset, 2005), which regulate plastic responses in life‐history traits such as reproductive timing (Huang et al., 2016; Wilczek et al., 2009). Consequently, short delays in emergence can be magnified into large differences in final biomass and reproduction (Burghardt et al., 2015; Donohue, 2005; Galloway & Burgess, 2009; Wilczek et al., 2009), especially under competitive conditions (Dyer et al., 2000; Kelly & Levin, 1997). For instance, plants with delayed emergence can have shortened vegetative growth and earlier reproduction at smaller plant sizes due to shorter life cycles, than early‐emerged plants (Zhou et al., 2005).

Plants germinated early have more time to acquire resources to grow for reproduction but may face an increased risk of mortality since stresses often occur earlier in the season such as drought, frost, or predation (Donohue et al., 2010; Lortie & Turkington, 2002; Verdu´ & Traveset, 2005). Most previous studies believed that early emergence benefits plant performance and survival (Abe et al., 2008; Afonso et al., 2014; Bianchi et al., 2019; Cogoni et al., 2013; Verdu´ & Traveset, 2005). The ecological significance of delayed emergence may thus be underestimated (Verdu´ & Traveset, 2005; Wu & Owen, 2014). However, late emergence can confer higher fitness benefits (Castro, 2006; Leverett et al., 2018; Wu & Owen, 2014), by contributing more to seed production, in comparison with the more contribution to the competition of the earlier germinants (Grundy, 2003). Therefore, both early and late emergence can have beneficial effects, selection may favor either early, intermediate, or late emergence (Donohue et al., 2010; Kalisz, 1986; Verdu´ & Traveset, 2005), depending on specific circumstances. The selection should be a combination of different abiotic and biotic factors (Verdu´ & Traveset, 2005), and different selections may be conflicting (Akiyama & Ågren, 2014). It suggests there might be an optimal time span for plant emergence, within which plants can maximize overall performance in unpredictable environments (Gremer et al., 2020; ten Brink et al., 2020). Unfortunately, we know little about the optimal emergence time of wild plant species and its influences on plant subsequent performance.

On the contrary, we know better about the growth strategies of plants emerged early or late, in terms of varying life‐history traits. For instance, spring‐germinated plants will have prolonged vegetative growth and life cycle with delayed reproduction, as a strategy of competitors (C), whereas summer‐germinating plants tended toward the strategy of ruderals (R) with compressed vegetative growth and earlier reproduction than early germinants (Zhou et al., 2005). Phenotypic plasticity, defined as the ability of a genotype to make adjustments morphologically and physiologically in response to different environmental conditions (Bradshaw, 1965), has been regarded as an important mechanism for plant adaptation to variable environments. How the plasticity in a number of morphological traits contributes to the life‐history strategies of plants in response to emergence timing has not been well documented. Phenotypic response at the whole‐plant level constitutes integrated responses of plant modules and characters (de Kroon et al., 2005), the local responses may be different and even contrary (Wang & Zhou, 2022). Shifts in emergence timing can cause cascading effects on different modules or traits and thus fitness (Gremer et al., 2020), and different local responses may interact to determine the final phenotype of plants. It is unclear how germination time cascading affects various traits and life history strategies (Verdu´ & Traveset, 2005).

Another important aspect should be the effects of plant ontogeny. Most studies on the effects of emergence timing have examined plant performance in morphological traits at the final growth stage (Afonso et al., 2014; Wang et al., 2006; Zhou et al., 2005), lacking the information on their dynamic changes or comparisons on plants of the same growth periods. For example, by comparing final performances, it appears that early germinants can perform better than late germinants. However, this may simply be because early germinants have a longer growth period than late ones, and the latter may accumulate greater biomass (grow more rapidly) than the former given the equal time for growth. Additionally, the pattern of biomass allocation varies with different stages due to allometric growth (Weiner, 2004), the responses of plants to emergence timing may largely depend on specific growth stages. Therefore, to address the effects of emergence timing requires the information on the dynamic pattern of morphological plasticity (ten Brink et al., 2020).

To better understand how plants respond to emergence timing via morphological plasticity at different growth stages, in relation to their life‐history strategies, we conducted a field experiment, with an annual weed species of *Abutilon theophrasti*, by subjecting plants to four emergence treatments and measuring a number of traits at different stages. We ask the following questions: (1) How plants respond to emergence timing via plasticity in mass and morphological traits? (2) Do these responses vary with different growth stages? and (3) How these responses contribute to plant strategies in dealing with variable emergence time?

## 2 MATERIALS AND METHODS

### 2.1 Study species

*Abutilon theophrasti* Medicus (Malvaceae) is an annual weedy species native to China and India but now spreads worldwide. Generally, its emergence period ranges from April to July, and it grows rapidly to a height of 1–1.5 m with stout stems, reaching reproductive maturity within 90 days, and completes its life cycle in about 5 months (McConnaughay & Coleman, 1999). It colonizes relatively nutrient‐rich habitats and has substantial plasticity in allocation, morphology, and architecture in response to varying environmental factors (McConnaughay & Bazzaz, 1992).

### 2.2 Experimental design

**FIGURE 1:**
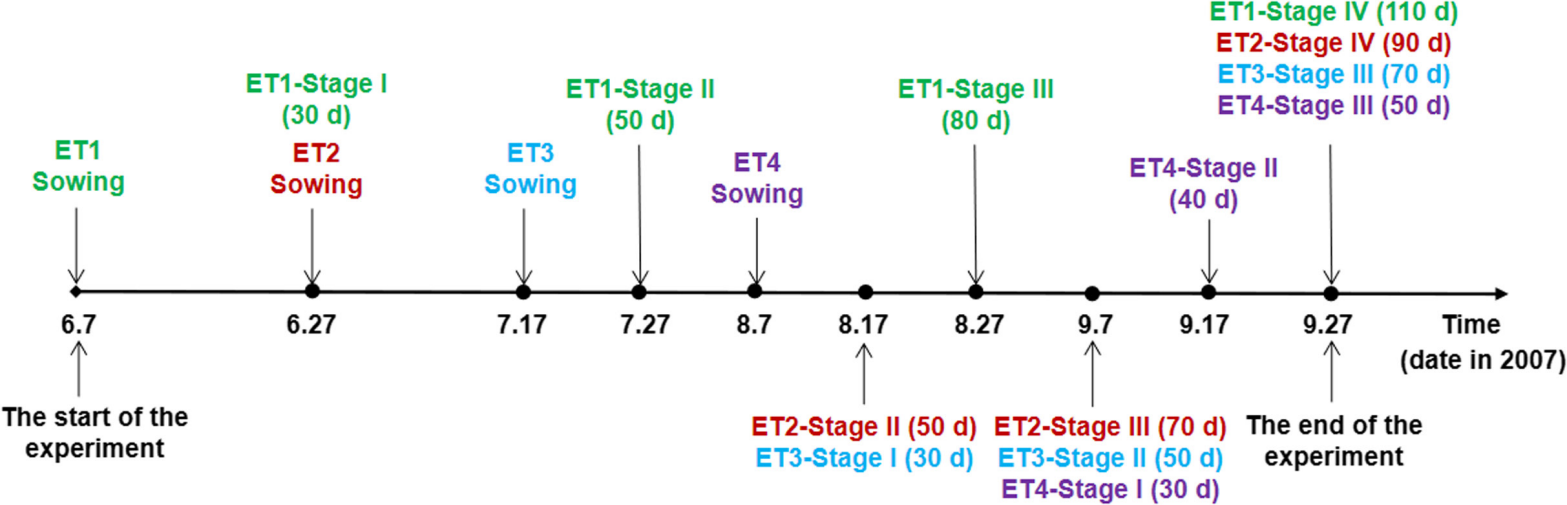
The timing of sowing and harvesting (stage I‐IV) with growth periods (days) for all treatments of emergence time (ET1‐4).

We conducted the field experiment in 2007 at the Pasture Ecological Research Station of Northeast Normal University, Changling, Jilin province, China (44°45′ N, 123°45′ E). The original soil of the experimental field (eolian sandy soil, pH = 8.3) at the station had been used annually for many years, thereby low in nutrients availability (organic C 3.1 mg kg^−1^, available N 21.0 mg kg^−1^, and available P 1.1 mg kg^−1^) during the growth season of 2007 (Zhao et al., 2010). Seeds of *A. theophrasti* were collected from local wild populations near the research station in late August 2006. All the seeds were dry and stored at −4°C till for use in the experiment. We applied a randomized block design, with emergence timing (ET) as the main factor, and block as the sub‐factor. The whole plot was divided into 12 2 × 3 m sub‐plots, which were randomly assigned with four ET treatments and three blocks. The sowing dates for the four ET treatments were June 7, June 27, July 17, and August 7, representing spring (ET1), late spring (ET2), summer (ET3), and late summer (ET4), respectively (Figure 1). The treatments accorded with the time range of emergence of *A. theophrasti* in its natural habitats in the northeast China. In the northeast China, spring usually ranges from April to June annually. Changchun locates in the northeast by north and spring is regarded to start in late April, summer starts in early July, autumn starts in mid‐August, and winter starts in mid‐October. However, the weather between April and May is often chilling with unpredictable precipitation. To avoid severe mortality of seedlings in the early season, we did not make the emergence of plants occur during this time span, instead made the plants emerging in early June as the treatment for spring emergence, and those emerging in late June as the treatment of late spring. Seeds of *A. theophrasti* were sown with an inter‐planting distance of 10 cm and most of them emerged 4 days after sowing. Seedlings were thinned at the four‐leaf stage and plots were hand‐weeded when necessary and watered regularly.

### 2.3 Data collection

For each treatment, we arranged three to four times of sampling, according to their growth stages and the lengths of the life cycle, and generally harvested them at the stages of vegetative growth, late vegetative or early reproductive growth, and middle to late reproductive growth, respectively (Table 1). For individuals that emerged in spring (ET1), we took an additional sample at their early vegetative growth stage as an extra harvest (EX), as a second reference (the first one is the vegetative growth stage) for comparison with the first samplings of other treatments, since the plants emerged early had a prolonged vegetative growth. At each harvest, five to six individual plants were randomly chosen from each plot, making a maximum total of 6 replicates × 3 blocks × 4 treatments × 3 harvests +6 replicates × 3 blocks × 1 treatment (SP) × 1 harvest = 234 samplings. For each individual plant, the following traits were measured (if applicable): main root length, diameter at the basal of the main root, length, and number of lateral roots (above or equal to 1 mm in diameter along the main root), the length of stem, diameter at the base of stem, petiole length, and angle, leaf number, lamina width (leaf size, abbreviations for all traits are in Table 2). For individuals from ET1 and ET4 treatments, some traits were unavailable for measurement at early growth stages due to small plant sizes. Each plant individual was then separated into roots, stems, petioles, laminas, reproductive modules, and branches (if there were any), oven‐dried at 75°C for 2 days and weighed. Reproductive modules consisted of flowers and fruits produced along the main stem and branches, and branches included the stems and leaves on branches. The total mass and mass allocation traits were calculated.

**TABLE 1:** The treatments of emergence time and the growth stages and periods at harvest in this study

Emergence time	Abbrev.	Sowing date	Growth stage	Sampling date	Growth period (days)	Final harvest
Spring	ET1	Jun. 7	I	Jun. 27	20	
			**II**	**Jul. 27**	**50**	
			**III**	**Aug. 27**	**80**	
			**IV**	**Sep. 27**	**110**	√
Late spring	ET2	Jun. 27	I	Jul. 27	30	NA
			**II**	**Aug. 17**	**50**	
			**III**	**Sep. 7**	**70**	
			**IV**	**Sep. 27**	**90**	√
Summer	ET3	Jul. 17	I	Aug. 17	30	
			**II**	**Sep. 7**	**50**	
			**III**	**Sep. 27**	**70**	√
Late summer	ET4	Aug. 7	I	Sep. 7	30	
			II	Sep.17	40	
			**III**	**Sep. 27**	**50**	√

*Note*. The bold font indicates the stage when plants were analyzed for effects of emergence time on mass and allocation traits at day 50, 70 (or 80), and final harvest. The data of the first sampling for ET2 were not available (NA) and not included in the analyses.

**TABLE 2:** Abbreviations for all the traits with units used in this study

Abbreviation	Trait	Unit
TM	Total mass	g
RM	Root mass	g
SM	Stem mass	g
PM	Petiole mass	g
LM	Lamina mass	g
REM	Reproductive mass	g
BM	Branch mass	g
RMR	Root mass ratio	/
SMR	Stem mass ratio	/
PMR	Petiole mass ratio	/
LMR	Lamina mass ratio	/
REMR	Reproductive mass ratio	/
BMR	Branch mass ratio	/
RL	Main root length	cm
RD	Main root diameter	mm
LRL	Lateral root length	cm
LRN	Lateral root number	/
SL	Stem length	cm
SD	Stem diameter	mm
PL	Petiole length	cm
PA	Petiole angle	o
LS	Lamina size	mm
LN	Leaf number	/

### 2.4 Statistical analysis

Statistical analyses were conducted using SAS statistical software (SAS Institute 9.0 Inc., 2002). All measured and calculated traits were used for analysis (Table 2). To minimize variance heterogeneity, all data were log‐transformed, except for petiole angles and branch angles (square root‐transformed), before statistical analysis. For plant total mass, we applied two‐way ANOVA to analyze the effects of emergence timing, sampling time and their interactions, and one‐way ANOVA to analyze the effects of emergence timing or sampling time within each or across all of the other treatments. Plant size (e.g., total mass) can have very significant effects on other traits, which may bias the effects of emergence time. Therefore, for all the other traits, we applied two‐way ANCOVA to evaluate the overall effects of emergence timing, sampling time and their interactions, and one‐way ANCOVAs for effects of emergence timing or sampling time within each or across all of the other treatments, with total mass as a covariate. For a given trait, the significant contribution of total biomass (plant size) to its variation in response to emergence timing indicates an occurrence of apparent plasticity (McConnaughay & Coleman, 1999). When effects of total mass were removed, the variation due to emergence timing in trait expression was an indication of true plasticity (Weiner, 2004). Multiple comparisons used the Least Significant Difference (LSD) method in the General Linear Model (GLM) program, which produced adjusted mean values and standard errors in one‐way ANCOVA.

## 3 RESULTS

**FIGURE 2:**
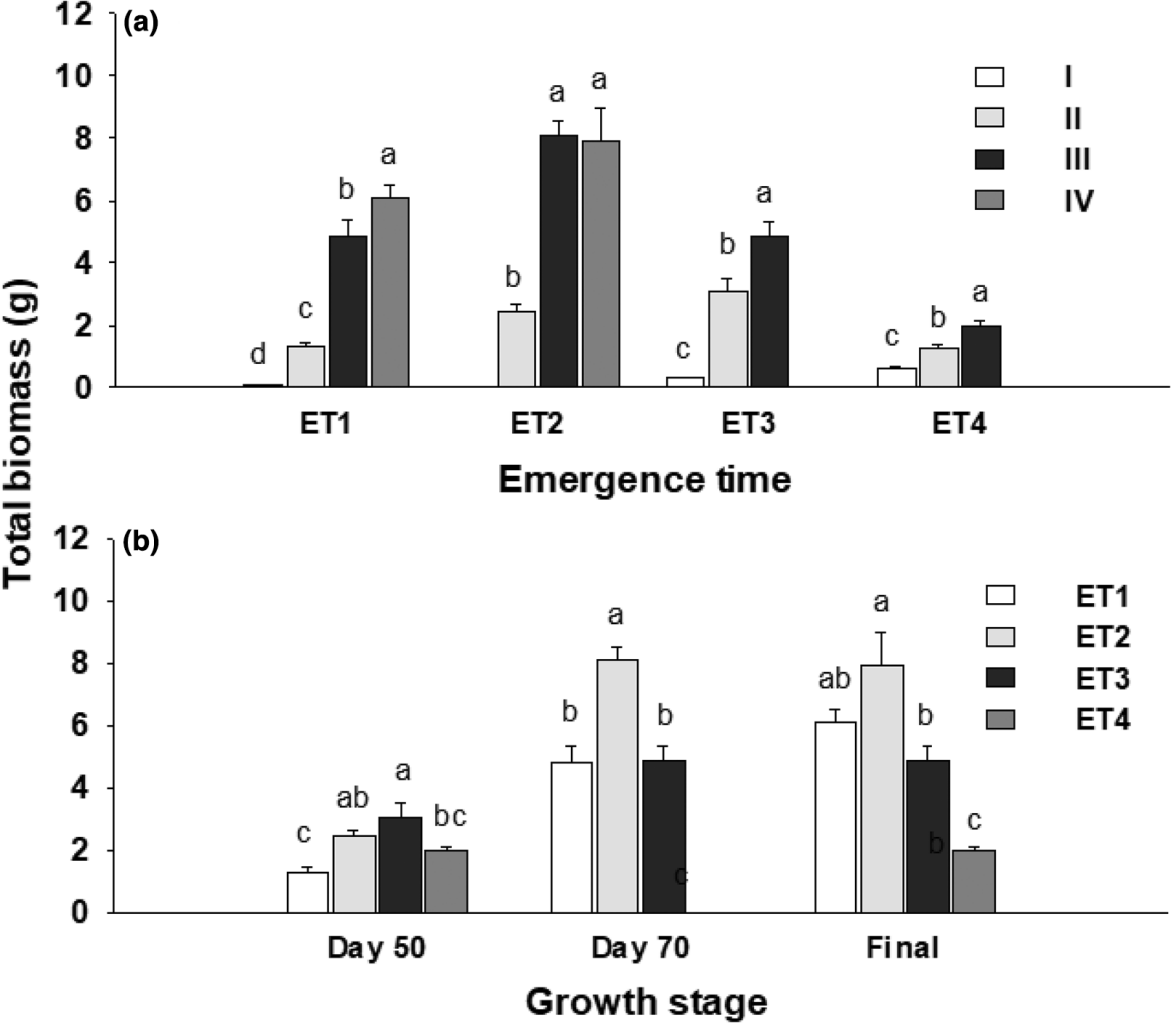
Mean total biomass (±SE) of plants that emerged in spring (ET1), late spring (ET2), summer (ET3), and late summer (ET4) at different growth stages (I‐IV; a) and compared at day 50, 70, and final harvest (b). Different letters indicate a significant difference between growth stages within emergence treatment (a), and between ET treatments for the same growth period (b; ANOVA, LSD, *p* < .05).

Emergence time, growth stage, and their interaction had significant effects on total mass (Table 3; Figure 2). Across all treatments of emergence time (ET), total mass increased as plants grew larger (ANOVA, F_GS_ = 36.40; df = 3, 215; *p* < .001; Figure 2a; Table S1). Plants that emerged in spring (ET1) used a long growth period to reach 6.10 ± 0.39 g of final total mass at day 110, whereas those that emerged in late spring (ET2) and summer (ET3) grew rapidly, reaching 8.08 ± 0.45 g and 4.85 ± 0.47 g of total mass within 70 days, which was higher (ANOVA, LSD, *p* < .001) or not lower than that of ET1 germinants (Figure 2). The plants that emerged in late spring (ET2) had the greatest total mass of all across both stages of day 50 and 70 (ANOVA, F_ET_ = 8.66; df = 3, 114; *p* < .001; Figure 2b; Table 4). Those emerged in late summer (ET4) had the lowest total mass of all at the final harvest (ANOVA, LSD, *p* < .001).

**TABLE 3:** *F*‐values from two‐way ANCOVA on all traits for effects of emergence timing (ET), growth stage (GS), and their interactions, with total mass (TM) as a covariate

Trait	N	TM (df = 1)	ET (df = 3)	GS (df = 3)	ET × GS (df = 6)
TM	216		82.71***	98.11***	13.39***
RM	216	196.30***	4.57***	6.76***	5.22**
SM	216	481.15***	54.02***	71.99***	19.374***
PM	202	54.45***	1.89	9.58***	4.28**
LM	216	152.08***	15.84***	27.45***	16.42***
REM	64	6.51*	0.25	2.98	‐‐
BM	165	360.83***	1.486	7.74***	0.44
RMR	216	13.10***	4.50***	5.68**	3.46**
SMR	216	38.45***	116.93***	81.84***	29.74***
PMR	202	0.58	47.64***	28.02***	0.79
LMR	216	8.69**	133.54***	200.96***	20.10***
REMR	165	51.31***	9.72***	9.59***	26.26***
BMR	64	0.94	0.35	2.82	–
RL	188	2.12	9.83***	15.41***	30.70***
RD	188	48.23***	32.89***	16.46***	4.83***
LRL	170	5.97*	9.66***	3.85***	2.84*
LRN	170	7.45*	0.84	3.90*	2.74*
SL	202	19.09***	88.96***	111.15***	20.77***
SD	202	35.98***	112.47***	35.02***	144.83***
PL	170	6.14*	0.38	0.63	0.10
PA	170	0.74	55.35***	50.42***	15.18***
LS	205	47.96***	18.66***	5.19**	19.64***
LN	170	1.45	17.53***	54.25***	19.23***

*Note*. N indicates the total number of individual plants measured for a given trait. Abbreviations for all traits were in Table 2.

* *p* < 0.05

** *p* < 0.01

*** *p* < 0.001.

**TABLE 4:** *F*‐values from one‐way ANCOVA on biomass and allocation traits for effects of emergence timing (ET), with total mass (TM) as a covariate, at day 50, 70, and the final harvest

Trait	Type	Day 50		Day 70		Final	
		TM (df = 1)	ET (df = 3)	TM (df = 1)	ET (df = 2)	TM (df = 1)	ET (df = 3)
TM	ANOVA		7.69***		14.42***		19.44***
RM	ANOVA		1.99		0.52		11.26***
	ANCOVA	2.01	0.70	69.11**	17.42***	99.55***	4.52**
SM	ANOVA		15.25***		20.28***		69.32***
	ANCOVA	219.02***	45.26***	85.73***	25.21***	162.90***	69.69***
PM	ANOVA		4.86**		6.04**		2.38
	ANCOVA	221.94***	7.24***	5.51*	1.06	120.50***	28.27***
LM	ANOVA		3.97*		9.61***		4.80**
	ANCOVA	685.62***	7.61***	12.32**	2.68	93.15***	34.07***
REM	ANOVA		12.96***		10.87***		12.62***
	ANCOVA	152.39***	15.41***	178.12***	4.69*	340.20***	14.03***
BM	ANOVA				0.50		0.26
	ANCOVA			2.58	0.18	3.90	0.99
RMR	ANOVA		1.87		19.01***		3.42*
	ANCOVA	41.32***	7.19***	0.15	11.35***	0.38	1.44
SMR	ANOVA		41.48***		41.41***		91.54***
	ANCOVA	1.13	41.36***	10.08**	48.23***	26.12***	125.20***
PMR	ANOVA		9.50***		0.80		119.45***
	ANCOVA	0.04	8.66***	1.27	1.43	0.28	68.25***
LMR	ANOVA		12.05***		0.80		220.79***
	ANCOVA	0.03	9.93***	17.70***	1.18	0.22	115.38***
REMR	ANOVA		11.37***		10.08***		41.66***
	ANCOVA	69.04***	12.11***	21.66***	11.96***	16.43***	34.66***
BMR	ANOVA				0.028		1.72
	ANCOVA			0.08	0.14	0.001	1.47

*Note*. Abbreviations for all traits were in Table 2.

* *p* < 0.05

** *p* < 0.01

*** *p* < 0.001.

**FIGURE 3:**
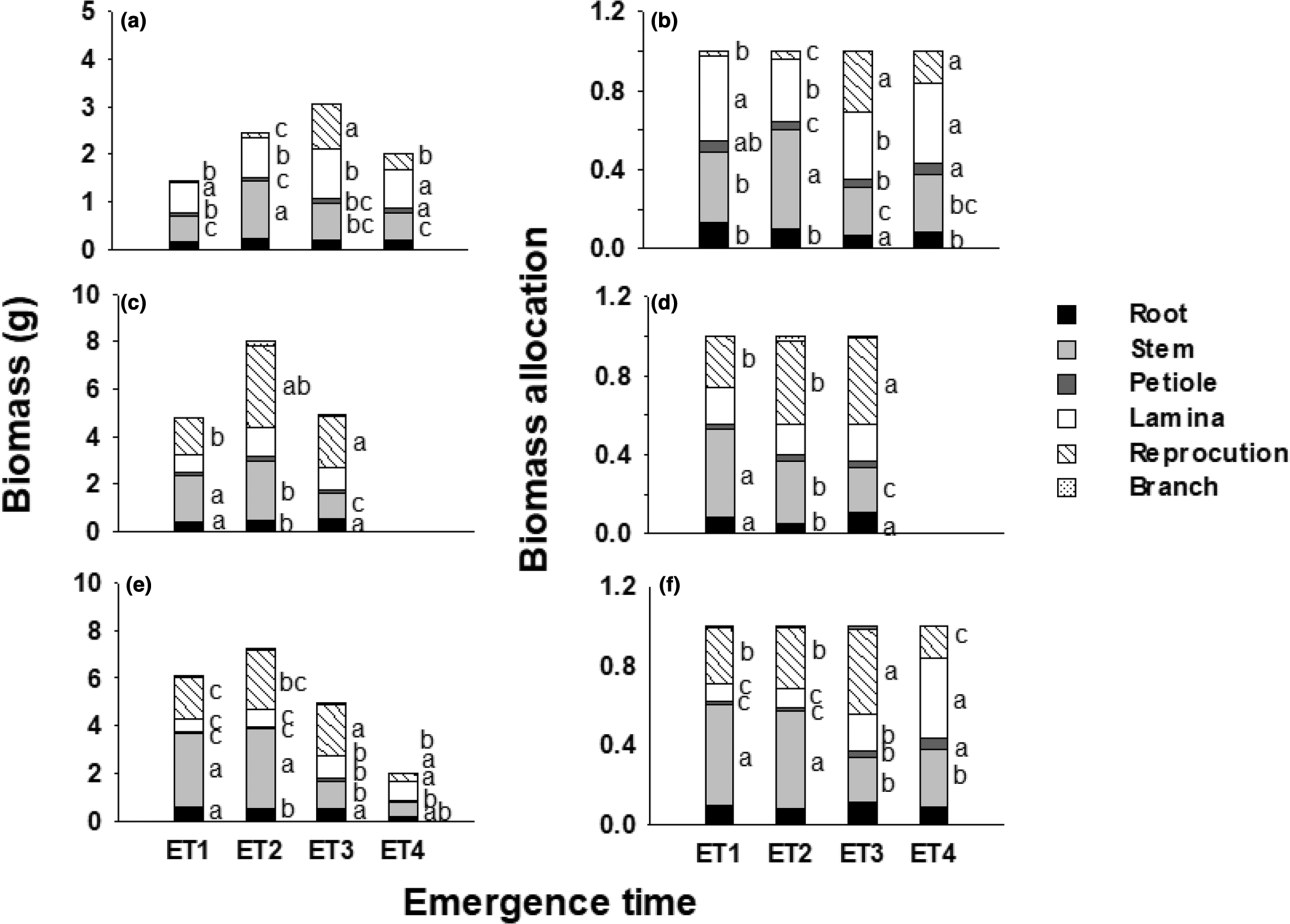
Mean values (±SE) of the mass (a, c, e) and allocation (b, d, f) of various organs for plants that emerged in spring (ET1), late spring (ET2), summer (ET3), and late summer (ET4) at day 50 (a, b), 70 (c, d), and the final (e, f) stages. Different letters indicate a significant difference between ET treatments within each stage (ANCOVA, LSD, *p* < .05).

**FIGURE 4:**
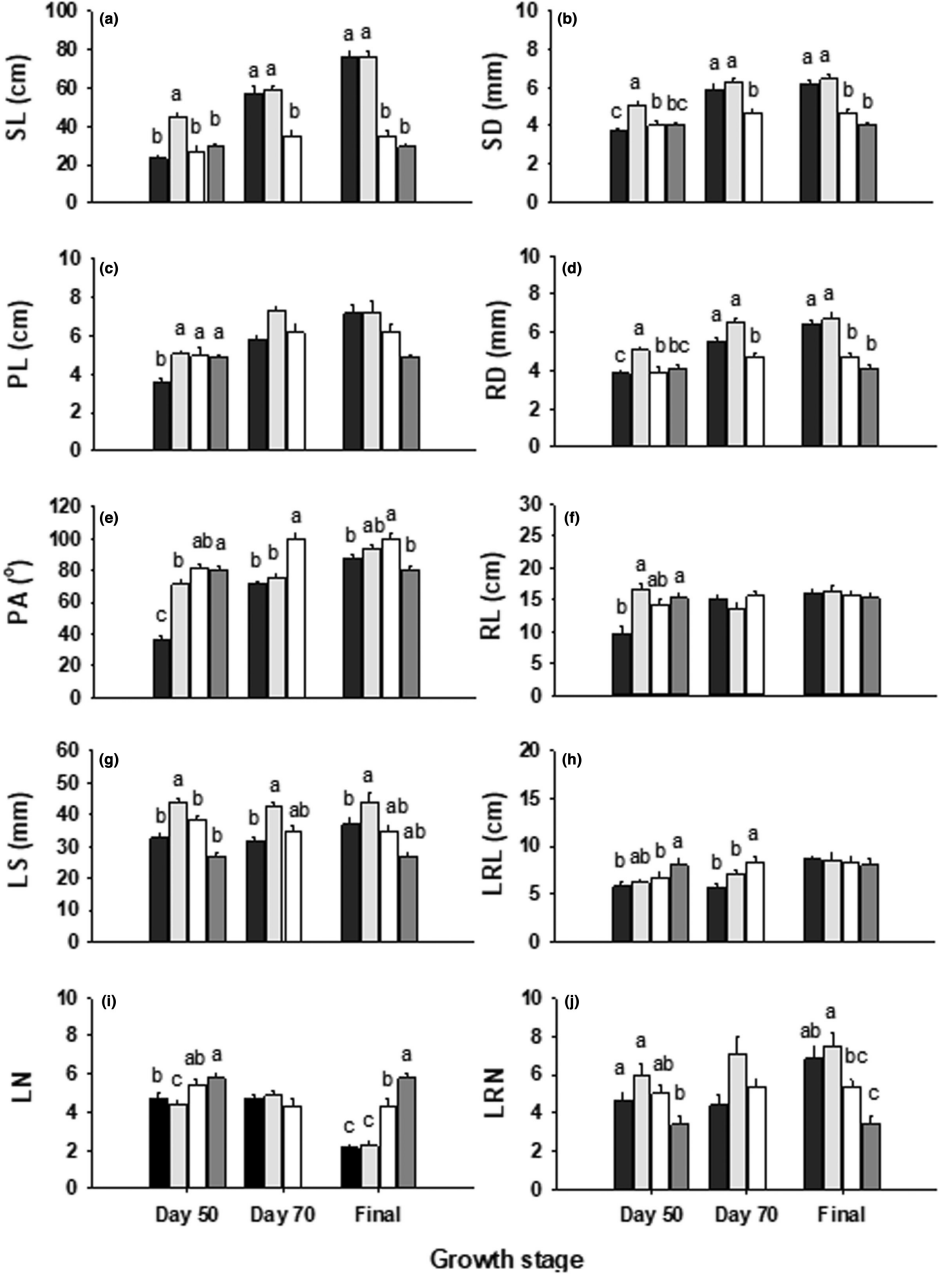
Mean values (±SE) of morphological traits for stem (a, b), leaf (c, e, g, i), and root (d, f, h, j) organs of plants that emerged in spring (ET1, black), late spring (ET2, light gray), summer (ET3, white), and late summer (ET4, dark gray) at day 50, 70, and final stages. Different lowercase letters indicate a significant difference between ET treatments within each stage (ANCOVA, LSD, *p* < .05). Abbreviations for all traits were in Table 2.

Effects of total mass accounted for a significant proportion of variation for most traits; after removing size effects, however, effects of emergence time, growth stage, and their interaction were still significant in most cases (Table 3; Figures 3 and 4). Across the stages of day 50, 70, and final harvest, ET3 germinants had the highest reproductive mass and allocation of all (ANCOVA, LSD, *p* < .05), and ET2 germinants had the greatest leaf size of all (*p* < .05; Figure 3; Figure 4g). For each stage, effects of emergence time on traits were also significant in most cases (Tables 4 and 5). On day 50, ET2 germinants had the highest stem mass and allocation of all (Table 3; Figure 3a,b; *p* < .05), with the longest and thickest stems and main roots, and the lowest leaf number (*p* < .05; Figure 4a,b,d,f); ET1 germinants performed lower in petiole length and angle, leaf number, main root length and lateral root length than ET4 (*p* < 0.05; Figure 4c,e,f,h,i). On day 50 and final harvest, leaf number increased with later emergence, but ET4 had the lowest lateral root number of all (Figure 4j). On day 70 and the final harvest, stem mass and allocation decreased with later emergence (*p* < .05; Figure 3c–f), and plants of ET1 and ET2 performed higher in stem length and diameter and root diameter than those of ET3 and ET4 (*p* < .01; Figure 4a,b,d), but ET3 had the greatest petiole angle of all (*p* < .001; Figure 4e). At the final harvest, leaf (petiole and lamina) mass and allocation increased with later emergence (*p* < .01; Figure 3e,f).

**TABLE 5:** *F*‐values from one‐way ANCOVA on morphological traits for effects of emergence timing (ET), with total mass (TM) as a covariate, at day 50, 70, and the final harvest

Trait	Type	Day 50		Day 70		Final	
		TM (df = 1)	ET (df = 3)	TM (df = 1)	ET (df = 2)	TM (df = 1)	ET (df = 3)
TM	ANOVA		33.73***		13.77***		21.39***
RL	ANOVA		13.66***		2.89		0.51
	ANCOVA	1.83	9.84***	2.96	0.36	1.53	0.013
RD	ANOVA		10.61***		18.76***		38.18***
	ANCOVA	56.27***	17.83***	0.74	10.40***	19.64***	14.59***
LRL	ANOVA		5.69**		10.16***		0.31
	ANCOVA	0.37	5.55**	2.03	11.10***	8.25**	0.74
LRN	ANOVA		6.18**		3.17		11.42***
	ANCOVA	3.89	4.99**	3.37	0.72	0.57	4.20**
SL	ANOVA		41.68***		30.31***		95.35***
	ANCOVA	25.22***	49.12***	1.30	24.29***	2.13	51.95***
SD	ANOVA		15.26***		17.59***		44.42***
	ANCOVA	68.11***	33.09***	0.17	13.27***	24.47***	17.11***
PL	ANOVA		29.33**		6.94**		8.29***
	ANCOVA	37.70***	7.68***	10.03**	1.44	46.38***	1.23
PA	ANOVA		66.51***		40.97***		7.91***
	ANCOVA	2.53	45.33***	0.03	35.39***	0.47	5.89**
LS	ANOVA		23.33***		14.37***		7.59***
	ANCOVA	19.92***	26.24***	9.12**	3.09	26.97***	2.03
LN	ANOVA		14.11***		1.29		52.98***
	ANCOVA	6.41*	10.60***	0.23	0.85	0.32	30.88***

*Note*. Abbreviations for all traits were in Table 2.

* *p* < 0.05.

** *p* < 0.01.

*** *p* < 0.001.

For all ET treatments, mass traits and most morphological traits increased over time (ANCOVA, LSD, *p* < .05; Table S1 and S2; Figure S1 and S2). In later stages, plants of all ET treatments had greater reproductive mass and allocation (*p* < .05; Figure S1), smaller lamina allocation (*p* < .01; Figure S1b,d,f,h), and longer stems and roots (Figure S2a,f); E1 and ET4 plants had greater stem allocation (*p* < .001; Figure S1b,h), and ET1 and ET2 had less leaves and ET4 had less lateral roots at the third stage than at earlier stages (*p* < .001; Figure S2i,j).

## 4 DISCUSSION

Plants of *Abutilon theophrasti* can germinate over a wide range of period in growth seasons. Natural selection may favor emergence at different times or a mixed, bet‐hedging strategy (Silvertown, 1988; Zhou et al., 2005). The plasticity in growth strategy may compete with bet‐hedging as a way to deal with environmental variation (Donaldson‐Matasci et al., 2013; Xue & Leibler, 2018), if environments can be predicted (ten Brink et al., 2020). For example, emergence timing can influence the strength and direction of selection on plant performance and response in growth and reproductive traits later in the life cycle (Donohue et al., 2005; Mercer et al., 2011; Weinig, 2000). Early emergence generally results in higher fecundity, but selection on survival may favor early, intermediate, or late emergence (Donohue et al., 2010; Kalisz, 1986; Verdu´ & Traveset, 2005). Different selections on emergence timing may be conflicting (Akiyama & Ågren, 2014), suggesting the existence of optimal emergence times for different species (Gremer et al., 2020; ten Brink et al., 2020). Our results showed that the optimal emergence time for *A. theophrasti* was in late spring, plants that emerged earlier or later had decreased performance to different extents. Responses of plants germinated at different times also revealed their different growth strategies: spring and late‐spring germinants (ET1 and ET2) had prolonged vegetative growth and life cycle, with extensive stem growth and delayed reproduction, indicating competitive strategy; plants with delayed emergence (ET3 and ET4) had compressed vegetative growth, with accelerated leaf growth, advanced reproduction, and shortened life cycle, indicating ruderal strategy (Grime, 1979; Hodgson, 1999; Zhou et al., 2005). In spite of disadvantages, plants with delayed emergence were still able to reduce the adverse environmental effects via plasticity in numerous traits.

### 4.1 Responses of plants emerged in late spring

Our results showed plants that germinated in late spring (ET2) performed the best in the final total mass of all, with the highest performance in morphological traits such as stem and root length and diameter, and leaf size but the lowest leaf number at day 50. Different emergence times can expose plants to varying environmental signals, and plants will perceive and transmit the signals of changing photoperiod and temperature (Zhou et al., 2005), to promote the time of emergence, growth, and reproduction to coincide with favorable conditions (Andrés & Coupland, 2012; Blackman, 2017). Early emergence can improve the performance and survival of plants (Abe et al., 2008; Afonso et al., 2014; Bianchi et al., 2019; Cogoni et al., 2013; Verdu´ & Traveset, 2005), by virtue of a long growth period in season (Donohue et al., 2010; Stratton, 1992) and no environmental hazards. Early germinants (ET1 and ET2) preferred stem growth to leaf and reproductive growth, as time was sufficient for them to accomplish reproduction before the end of the growth season. The extensive vegetative growth then became an advantage over later germinants, especially in dense populations (Miller et al., 1994; Orrock & Christopher, 2010). The inter‐plant distance (10 cm) at sowing in this study may cause competition among plants, inducing additional stem elongation, especially in spring and late‐spring germinants. Analogous studies may provide further evidence by growing plants with different emergence times at lower densities.

### 4.2 Responses of plants emerged in spring

Plants that germinated earlier in spring (ET1) also had longer vegetative growth and life cycle but did not perform better than late‐spring germinants. In the northeast China, the climate during April and May is usually chilling (although the period is called spring), with unpredictable precipitation and frequent drought, which may cause mortality (Weekley et al., 2007). Despite the importance of early establishment (Miller et al., 1994), plants emerged early may thereby experience a higher risk of mortality due to seasonal hazards such as pathogens, predation, and desiccation (Donohue, 2014; Jones & Sharitz, 1989; Mercer et al., 2011; Rice, 1990). It suggests spring germinants may adapt to subsequent environments with the strategy as stress tolerators, in comparison with the strategies of competitors and ruderals for late‐spring germinants and later germinants, respectively (Zhou et al., 2005). Besides, they also had shorter and more‐vertical petioles, with thinner stems, thinner and shorter roots at day 50, resembling the typical “shade avoidance” response (Wang et al., 2017; Wang & Zhou, 2022), implying they experienced stronger intraspecific competition than those germinated in late spring and summer. Limited resources due to the competition may decelerate the growth of early seedlings, reducing the differences between them and late seedlings (Verdu´ & Traveset, 2005). Consequently, the spring plants did not outperform late‐spring plants, despite the length of growth period allows sufficient vegetative and reproductive growth. Nevertheless, they should still be able to compete with those germinated in summer and late summer (ET3 and ET4) with greater plant sizes (Bianchi et al., 2019).

### 4.3 Responses of plants with delayed emergence

In contrast, we found plants that emerged in summer (ET3) had decreased stem mass allocation but increased leaf mass allocation than late‐spring germinants, and the greatest reproductive mass allocation of all (Hartzler et al., 2004; Wu & Owen, 2014), and accumulated the same or even higher total mass as spring germinants within the same growth period (Zhou et al., 2005). Later emergence could promote resource partitioning (Leverett et al., 2018), or higher efficiencies of resource allocation (Wu & Owen, 2014). Plants with delayed emergence may give the priority to leaf and reproductive growth, avoiding extensive stem growth, for the completion of entire life cycles in unfavorable conditions (Sultan, 2000; Zhou et al., 2005). Plants with a further delay of emergence into late summer (ET4), however, performed the lowest in total mass and reproduction due to insufficient time for completing the life cycle, in spite of dramatically accelerated leaf growth. The delay of emergence may be very costly to fitness (Metcalf et al., 2003; Tuljapurkar, 1990) if it leads to an incomplete life cycle. Late‐summer germinants produced flowers, without enough time for fruit shaping and ripening.

Nevertheless, our results showed that plants with delayed emergence had increased leaf mass allocation and leaf number, and canalized petiole lengths, angle, and leaf size. Meanwhile, they also had canalized root mass allocation and root length, with decreased stem and root diameter and lateral root number. In contrast, in response to increased density, plants had decreased performance in leaf traits such as leaf number in infertile soil, but had canalized performance in more leaf traits in fertile soil (Wang et al., 2017); they also had decreased lateral root length in both soil conditions (Wang et al., 2021). The number of leaves or lateral roots and stem and root diameters may indicate the growth rate of modules, while the length or size (or area) characters probably reflect the restrictive effects of environmental conditions. For example, for late germinants, the increased leaf number thus suggested accelerated leaf growth, and decreased lateral root number was indicative of decelerated root growth, while decreased leaf number and increased lateral root number indicated slow leaf growth and fast root growth respectively for early germinants. When the time for growth is limited due to delayed emergence, plants may prefer leaf growth to stem or root growth, for completing the reproductive task within the shorter lifespan. Meanwhile, no decrease in the lengths of petioles and main and lateral roots with delayed emergence indicated that the resource availability did not limit plant growth. For plants under competition, however, in addition to induced drastic stem elongation, the extension of root or petiole length will more likely be restricted by limited space or resource availability, but leaf growth can be canalized when abiotic conditions are better (or decreased in poor abiotic conditions).

Limited resources (including space and time) can have both suppressive and facilitative effects (stimulating the additional growth of some modular traits) simultaneously, leading to complex effects on plants (Wang et al., 2017, 2021). For the increase in density, the suppressive effects may function through reducing the growth rate of modules (especially in deficiency of resources), while active effects through inducing responsive mechanisms for self‐adjustment. Comparatively, positive effects of delayed emergence may work through promoting the growth rate of modules, with the cost of decreased growth in other modules as negative effects.

## 5 CONCLUSIONS

Our results showed that the optimal emergence time was in late spring. Plants that emerged in late spring can maximize their growth potential in relatively favorable conditions, but plants with either advanced or delayed emergence are able to adapt to subsequent environments via plasticity in a number of traits. Plants emerged early (ET1 and ET2) preferred stem growth to leaf and reproductive growth, as they had sufficient time for accomplishing reproduction before the end of the growth season. Spring germinants did not outperform late‐spring ones, but they were still able to compete with those emerged late (ET3 and ET4) with greater plant sizes.

In contrast, plants with delayed emergence (ET3 and ET4) preferred leaf and reproductive growth to stem growth for the completion of entire life cycles. Consequently, plants emerged in summer (ET3) had the greatest reproductive allocation of all, whereas late‐summer germinants (ET4) performed the lowest in total mass and reproduction due to an incomplete life cycle. The number of leaves or lateral roots and stem and root diameters may indicate the growth rate of modules, while the length or size (or area) characters may reflect any restriction by environmental conditions. Low availability of resources (or space/time) can have both suppressive and facilitative effects. The delay of emergence may have positive effects through accelerating leaf growth (indicated by increased leaf number) at the cost of the growth of other organs (negative effects), while canalizing length or diameter characters without limitation of abiotic conditions.

This study has provided an insight into how the plasticity in various allocation and morphological traits contributes to the growth strategies of plants to deal with the complex biotic environmental changes such as delayed emergence, in the hope of better understanding the evolution of life‐history traits in plants.

## CONFLICT OF INTEREST

The authors have no conflict of interest to declare.

## Supporting information


Table S1
Table S2
Figure S1
Figure S2

## Data Availability

The data that support the findings of this study are openly available in: https://doi.org/10.5061/dryad.n8pk0p2w7
