# Supplementary material for: Dynamic morphological plasticity in response to emergence timing in Abutilon theophrasti (Malvaceae)
Source: Plant Environ Interact. 2022 Jun 18;3(3):118–29. doi: 10.1002/pei3.10084 (PMC10168065; doi:10.1002/pei3.10084)
Supplement: Supplementary file 1 — Table S1 Table S2 Figure S1 Figure S2 [file PEI3-3-118-s001.docx]

**Supplementary Materials**

**Table S1** *F*-values from one-way ANCOVA on biomass and allocation traits for effects of growth stage (GS) , with total mass (TM) as a covariate. Abbreviations for all traits are in Table 2. * *p* < 0.05, ** *p* < 0.01, *** *p* < 0.001.

| Trait | Type | ET1 | | ET2 | | ET3 | | ET4 | |
| --- | --- | --- | --- | --- | --- | --- | --- | --- | --- |
|  |  | TM  (df = 1) | GS  (df = 3) | TM  (df = 1) | GS  (df = 2) | TM  (df = 1) | GS  (df = 2) | TM  (df = 1) | GS  (df = 2) |
| TM | ANOVA |  | 58.73*** |  | 25.50*** |  | 46.30*** |  | 64.97*** |
| RM | ANOVA |  | 26.76*** |  | 13.74*** |  | 78.13*** |  | 15.71*** |
|  | ANCOVA | 98.02*** | 1.39 | 149.50*** | 13.96*** | 14.16*** | 13.83*** | 12.09** | 0.35 |
| SM | ANOVA |  | 91.02*** |  | 34.62*** |  | 37.38*** |  | 98.75*** |
|  | ANCOVA | 195.77*** | 16.58*** | 84.97*** | 27.66*** | 205.25*** | 0.74 | 176.01*** | 16.38*** |
| PM | ANOVA |  | 5.66** |  | 6.82** |  | 25.45*** |  | 32.62*** |
|  | ANCOVA | 19.21*** | 6.40** | 7.44** | 3.86* | 160.80*** | 14.52*** | 0.008 | 4.59* |
| LM | ANOVA |  | 24.26*** |  | 10.37*** |  | 22.46*** |  | 21.88*** |
|  | ANCOVA | 15.70*** | 16.78*** | 32.44*** | 16.01*** | 210.15*** | 33.81*** | 35.26*** | 14.12*** |
| REM | ANOVA |  | 15.90*** |  | 21.73*** |  | 7.35* |  | 21.39*** |
|  | ANCOVA | 144.26*** | 3.28* | 203.47*** | 2.44 | 4.54* | 22.08*** | 14.98** | 1.02 |
| BM | ANOVA |  |  |  | 1.79 |  |  |  |  |
|  | ANCOVA | - | - | 2.15 | 2.35 | - | - | - | - |
| RMR | ANOVA |  | 8.08*** |  | 31.67*** |  | 3.54* |  | 3.47* |
|  | ANCOVA | 0.027 | 5.31** | 1.17 | 13.10*** | 31.55*** | 19.07*** | 0.016 | 0.88 |
| SMR | ANOVA |  | 51.56*** |  | 28.08*** |  | 15.97*** |  | 79.22*** |
|  | ANCOVA | 7.26** | 29.01*** | 26.32*** | 25.20*** | 0.42 | 3.87* | 0.059 | 23.41*** |
| PMR | ANOVA |  | 41.82*** |  | 9.12*** |  | 17.29*** |  | 8.21** |
|  | ANCOVA | 4.88* | 9.77*** | 0.083 | 7.93** | 0.16 | 9.63*** | 4.25* | 8.61** |
| LMR | ANOVA |  | 306.72*** |  | 51.65*** |  | 189.01*** |  | 172.06*** |
|  | ANCOVA | 18.16*** | 80.45*** | 0.12 | 25.98*** | 0.57 | 57.21*** | 2.64 | 31.13*** |
| REMR | ANOVA |  | 33.68*** |  | 79.59*** |  | 13.30** |  | 12.65** |
|  | ANCOVA | 30.45*** | 3.06 | 26.83*** | 26.55*** | 0.28 | 25.61*** | 1.25 | 1.72 |
| BMR | ANOVA |  |  |  | 11.44*** |  |  |  |  |
|  | ANCOVA | - | - | 0.26 | 2.64 | - | - | - | - |

**Table S2** *F*-values from one-way ANCOVA on morphological traits for effects of growth stage (GS) , with total mass (TM) as a covariate. Abbreviations for all traits are in Table 2. * *p* < 0.05, ** *p* < 0.01, *** *p* < 0.001.

| Trait | Type | ET1 | | ET2 | | ET3 | | ET4 | |
| --- | --- | --- | --- | --- | --- | --- | --- | --- | --- |
|  |  | TM  (df = 1) | GS  (df = 3) | TM  (df = 1) | GS  (df = 2) | TM  (df = 1) | GS  (df = 2) | TM  (df = 1) | GS  (df = 2) |
| TM | ANOVA |  | 81.42*** |  | 28.73*** |  | 68.71**** |  | 61.12*** |
| RL | ANOVA |  | 17.91*** |  | 6.08** |  | 15.29*** |  | 197.47*** |
|  | ANCOVA | 0.80 | 3.43* | 1.26 | 6.16** | 0.82 | 5.86** | 0.001 | 88.03*** |
| RD | ANOVA |  | 38.41*** |  | 19.08*** |  | 74.66*** |  | 45.67*** |
|  | ANCOVA | 10.80** | 4.47* | 44.97*** | 1.21 | 0.13 | 18.78*** | 43.46*** | 3.78* |
| LRL | ANOVA |  | 29.85*** |  | 6.53** |  | 15.49*** |  | 2.56 |
|  | ANCOVA | 1.67 | 24.62*** | 10.72** | 3.30* | 2.24 | 1.30 | 1.51 | 0.19 |
| LRN | ANOVA |  | 5.61** |  | 1.08 |  | 0.13 |  | 15.82*** |
|  | ANCOVA | 5.65* | 3.32* | 3.89 | 0.23 | 0.43 | 0.12 | 1.93 | 15.82*** |
| SL | ANOVA |  | 177.97*** |  | 49.68*** |  | 42.01*** |  | 94.16*** |
|  | ANCOVA | 56.59*** | 21.59*** | 2.75 | 30.57*** | 5.67* | 22.50*** | 25.42*** | 11.53*** |
| SD | ANOVA |  | 119.38*** |  | 16.44*** |  | 67.85*** |  | 248.86*** |
|  | ANCOVA | 4.25* | 20.81** | 57.07*** | 1.14 | 0.018 | 14.68*** | 3.71 | 148.26*** |
| PL | ANOVA |  | 33.19*** |  | 14.83*** |  | 0.44 |  | 26.09*** |
|  | ANCOVA | 6.81* | 5.21** | 37.80*** | 1.25 | 0.39 | 0.009 | 29.15*** | 3.47 |
| PA | ANOVA |  | 129.89*** |  | 19.28*** |  | 58.06*** |  | 2.64 |
|  | ANCOVA | 0.046 | 42.47*** | 0.39 | 15.90*** | 0.032 | 13.96*** | 3.44 | 0.008 |
| LS | ANOVA |  | 47.85*** |  | 0.23 |  | 38.70*** |  | 51.55*** |
|  | ANCOVA | 6.53* | 19.35*** | 12.50** | 4.25* | 26.25*** | 2.82 | 0.45 | 17.91*** |
| LN | ANOVA |  | 60.62*** |  | 47.57*** |  | 21.26*** |  | 3.77 |
|  | ANCOVA | 1.13 | 51.52*** | 144.38*** | 45.57*** | 0.84 | 11.81*** | 0.43 | 3.58 |

**Fig. S1** Mean values (±SE) of the mass (A, C, E, G) and allocation (B, D, F, H) of various organs for plants that emerged in spring (ET1; A, B), late spring (ET2; C, D), summer (ET3; E, F) and late summer (ET4; G, H) at different growth stages (I~IV). Different lowercase letters indicate significant difference between growth stages within emergence treatment (ET), different uppercase letters indicate significant difference between ET treatments for each stage (ANOVA, LSD, *p* < 0.05).


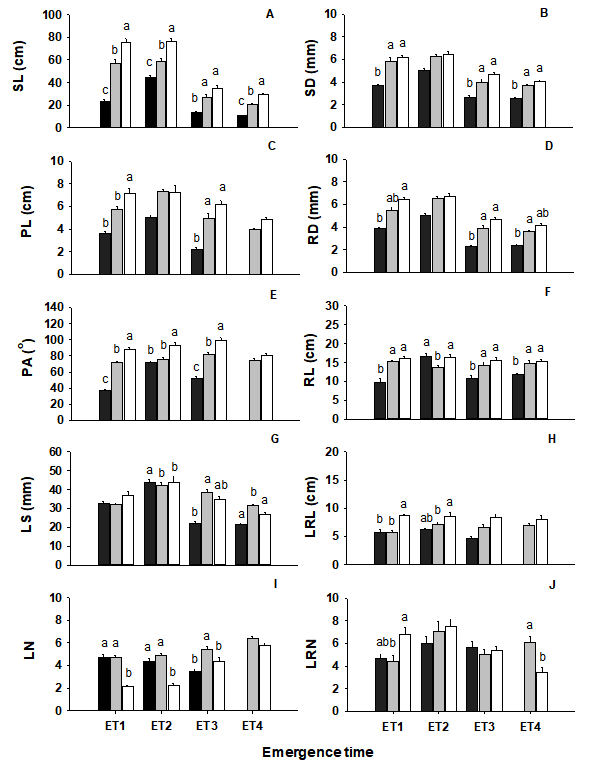


**Fig. S2** Mean values (±SE) of morphological traits for stem (A, B), leaf (C, E, G, I) and root (D, F, H, J) organs of plants that emerged in spring (ET1), late spring (ET2), summer (ET3) and late summer (ET4) at three growth stages of I or II (black), II or III (gray) and III or IV (white), with Stage II~IV for ET1 and ET2, and Stage I~III for ET3 and ET4. Different lowercase letters indicate significant difference between growth stages within each emergence treatment (ET; ANOVA, LSD, *p* < 0.05). Abbreviations for all traits were in Table 2.
